# Magnetic Resonance Perfusion-Weighted Imaging in Predicting Hemorrhagic Transformation of Acute Ischemic Stroke: A Retrospective Study

**DOI:** 10.3390/diagnostics13223404

**Published:** 2023-11-08

**Authors:** Ming Li, Yifan Lv, Mingming Wang, Yaying Zhang, Zilai Pan, Yu Luo, Haili Zhang, Jing Wang

**Affiliations:** 1 Department of Radiology, Ruijin Hospital, Shanghai Jiao Tong University School of Medicine, Shanghai 200025, China; mingli@tongji.edu.cn (M.L.); zilaipanlilly@rjh.com.cn (Z.P.); 2 Department of Radiology, Shanghai Fourth People’s Hospital, School of Medicine, Tongji University, Shanghai 200434, China; lv777evvan@tongji.edu.cn (Y.L.); wangmingming@tongji.edu.cn (M.W.); 2280314@tongji.edu.cn (Y.Z.); 1905219@tongji.edu.cn (Y.L.); 3 Southeast University Hospital, Southeast University, Nanjing 210096, China

**Keywords:** acute ischemic stroke, hemorrhagic transformation, magnetic resonance imaging, perfusion-weighted imaging

## Abstract

Hemorrhagic transformation (HT) is one of the common complications in patients with acute ischemic stroke (AIS). This study aims to investigate the value of different thresholds of Tmax generated from perfusion-weighted MR imaging (PWI) and the apparent diffusion coefficient (ADC) value in the prediction of HT in AIS. A total of 156 AIS patients were enrolled in this study, with 55 patients in the HT group and 101 patients in non-HT group. The clinical baseline data and multi-parametric MRI findings were compared between HT and non-HT groups to identify indicators related to HT. The optimal parameters for predicting HT and the corresponding cutoff values were obtained using the receiver operating characteristic curve analysis of the volumes of ADC < 620 × 10^−6^ mm^2^/s and Tmax > 6 s, 8 s, and 10 s. The results showed that the volumes of ADC < 620 × 10^−6^ mm^2^/s and Tmax > 6 s, 8 s, and 10 s in the HT group were all significantly larger than that in the non-HT group and were all independent risk factors for HT. Early measurement of the volume of Tmax > 10 s had the highest value, with a cutoff lesion volume of 10.5 mL.

## 1. Introduction

Acute ischemic stroke (AIS) is a leading cause of death and disability globally, resulting in high financial burden for families and society [1]. Hemorrhagic transformation (HT) is one of the common complications in patients with AIS and was first reported by Brain R. Ott and his colleagues [2]. HT refers to newly developed hemorrhage converted from bland infarction, detected by follow-up radiological examinations. It may occur in natural course or after reperfusion therapy, including thrombolysis and thrombectomy. The intravenous thrombolysis with recombinant tissue plasminogen activator (rt-PA) or intra-arterial thrombolytic therapy, as well as mechanical thrombectomy, improves clinical outcomes but increases the incidence of HT [3,4,5]. It is reported that HT occurs in 30–40% of AIS patients, with about 3% fatal intracerebral hemorrhage [6,7]. The prevention of HT is important for safe reperfusion therapy. Therefore, predicting patients with AIS who are at risk of developing HT is of critical significance.

Multiple studies have shown that magnetic resonance imaging (MRI) is valuable in predicting HT after AIS [8,9,10,11,12,13,14,15]. It has been reported that the assessments of parenchymal enhancement [9], apparent diffusion coefficient (ADC) values [10,11], fluid-attenuated inversion recovery (FLAIR) hyperintensity changes [12], microbleeds [13], regional cerebral blood volume [14], and T2*-permeability [15] may be useful for predicting HT. Recent research using machine learning of clinical information, multi-parameters diffusion-weighted imaging (DWI), and perfusion-weighted imaging (PWI) have been suggested as potential markers in the early prediction of HT [16,17]. PWI offers multiple parameters of cerebral blood volume (CBV), cerebral blood flow (CBF), mean transit time (MTT), time to peak (TTP), and time to maximum of the residual function (Tmax) that are linked to AIS [18].

Tmax has been shown to be a valuable parameter in evaluating the volume and location of the penumbra [19,20,21]. Tmax delays of >6 s (Tmax > 6 s) are optimal for the identification of penumbra, and the reperfusion of Tmax > 6 s lesions is associated with the neurological recovery of AIS patients [20,21]. Severe hypoperfusion has been shown to be related with severe blood–brain barrier (BBB) breakdown, whereas severe BBB leakage relates with higher HT [22]. Tmax with delays >8 s and 10 s indicate lesions with more severe hypoperfusion and relate to poor outcomes after reperfusion, especially when Tmax > 10 s [23]. In this study, we hypothesize that HT would be positively related with the volume of the high threshold of Tmax. If the optimal cutoff values of certain Tmax thresholds were found to be associated with the HT complication of AIS, this would potentially increase the clinical value of PWI in the assessment of AIS patients and guide clinical treatment, especially for those being considered for reperfusion therapy.

Despite the previous studies focusing on the value of Tmax on penumbra, final infarct size, and clinical outcome prediction [19,20,23,24,25], little research was dedicated to exploring the value of Tmax on the HT of AIS with a large patient size. DWI and PWI are the two commonly used MRI sequences for the evaluation of AIS patients in clinic. The parameters of the volumes of ADC < 620 × 10^−6^ mm^2^/s and Tmax > 6 s, 8 s, and 10 s are widely used and automatically generated from the Rapid Processing of Perfusion and Diffusion (RAPID) software (version 5.1, iSchemaView). Therefore, in this study, other than analyzing the potential predictors of clinical information, including age, gender, history of hypertension, diabetes mellitus, atrial fibrillation, time from symptom onset to first MRI, stroke severity of the National Institutes of Health Stroke Scale (NIHSS) score at admission, and reperfusion therapy, we aimed to investigate the correlation of different thresholds of Tmax (>6 s, >8 s, and >10 s) generated from PWI-MRI, as well as ADC values less than 620 × 10^−6^ mm^2^/s from DWI, with HT in AIS patients.

## 2. Materials and Methods

This retrospective study was approved by the institutional review board of Shanghai Fourth People’s Hospital Affiliated to Tongji University School of Medicine. Baseline data on demographic and clinical information, including the NIHSS score, were collected at admission. Written informed consent was obtained from each patient or from their designated representatives.

### 2.1. Patient Sample

We retrospectively reviewed all consecutive patients from January 2016 to June 2022 at Shanghai Fourth People’s Hospital Affiliated to Tongji University School of Medicine who were suspected to have AIS. Inclusion criteria for this study were: 1. Patients were admitted within 24 h from symptom onset; 2. patients received a non-contrast head CT scan immediately after arriving at the hospital to rule out intracranial hemorrhage and an emergency MRI with DWI and PWI within 1 h after admission; 3. patients had definite AIS manifestations on the MRI; and 4. patients received a follow-up CT or MRI within 14 days. Patients who were found with cerebral vascular malformation, brain tumors, and hemorrhage at the first brain CT or MRI were excluded. According to the results of the patients’ 14-day follow-up with CT or MRI, patients were categorized into the HT and non-HT groups.

### 2.2. MR Protocol

All MRI scans were performed with the 1.5 Tesla MRI system (Avanto, Siemens Healthcare, Erlangen, Germany) using a standard 20-channel head coil. The imaging sequences for each patient included T1-weighted imaging (T1WI), T2-weighted imaging (T2WI), FLAIR imaging, DWI, three-dimensional time-of-flight MR angiography (TOF-MRA), and dynamic susceptibility-weighted contrast-enhanced PWI. Imaging parameters for T1WI were as follows: repetition time (TR) = 1900 ms; echo time (TE) = 20 ms; field of view (FOV) = 220 × 220 mm; matrix size = 256 × 256; and slice thickness = 5 mm. Imaging parameters for T2WI were: TR = 5000 ms; TE = 125 ms; FOV = 220 × 220 mm; matrix size = 320 × 320; and slice thickness = 5 mm. Imaging parameters for FLAIR were: TR = 8500 ms; TE = 150 ms; FOV = 220 × 220 mm; matrix size = 320 × 320; and slice thickness = 5 mm. Imaging parameters for DWI were: TR = 3600 ms; TE = 100 ms; FOV = 220 × 220 mm; matrix size = 128 × 128; and slice thickness = 5 mm. Imaging parameters for PWI were: TR = 1520 ms; TE = 32 ms; FOV = 220 × 220 mm; matrix size = 128 × 128; and slice thickness = 5 mm. Imaging parameters for TOF-MRA were: TR = 21 ms; TE = 3.69 ms; FOV = 200 × 181 mm; matrix size = 303 × 384; and slice thickness = 0.6 mm. Gadopentetate dimeglumine (Gd-DTPA) was used at a dose of 0.2 mmol/kg for PWI imaging, with an injection rate of 4 mL per second.

### 2.3. Image Process

All MRI images were transferred to the picture archiving and communication system for image review and diagnosis. Rapid Processing of Perfusion and Diffusion (RAPID) software (version 5.1, iSchemaView) was used to automatically process the DWI and PWI data. The ADC map was used for the volumetric measurement of the ischemic core (with an ADC value < 620 × 10^−6^ mm^2^/s). Maps of Tmax > 6 s, 8 s, and 10 s were analyzed, respectively.

### 2.4. Statistical Analysis

All statistical analyses were performed using SPSS software (version 23.0, IBM Corp., Chicago, IL, USA, 2019). The level of significance was set as *p* < 0.05. Measurement information that conformed to normal distribution was expressed as the mean ± standard deviation, and the comparison between two groups was performed using the independent samples *t*-test. Measures that were not normally distributed were expressed as medians (interquartile ranges, IQRs), and the Mann–Whitney U test was used for comparisons between two groups. Count data were expressed as frequencies (percentages), comparisons between two groups of count data were performed using the chi-square test, and the Fisher exact test was used if necessary. Independent risk factors for HT were screened by multivariate logistic regression analysis with HT as the dependent variable (HT was assigned a value of 1, and non-HT was assigned a value of 0), and the items with statistical difference obtained by univariate analysis were used as independent variables for multivariate logistic regression analysis to further screen out the independent risk factors. Then, the odds ratio (OR) value, 95% confidence interval (CI), and *p*-value were calculated. The area under curve (AUC) of the four MRI parameters for predicting HT was analyzed using the receiver operating characteristic (ROC) curve, and the threshold values corresponding to the maximum Youden index were used to calculate the sensitivity, specificity, and accuracy.

## 3. Results

### 3.1. Demographic and Patient Characteristics in HT and Non-HT Groups

**Figure 1:**
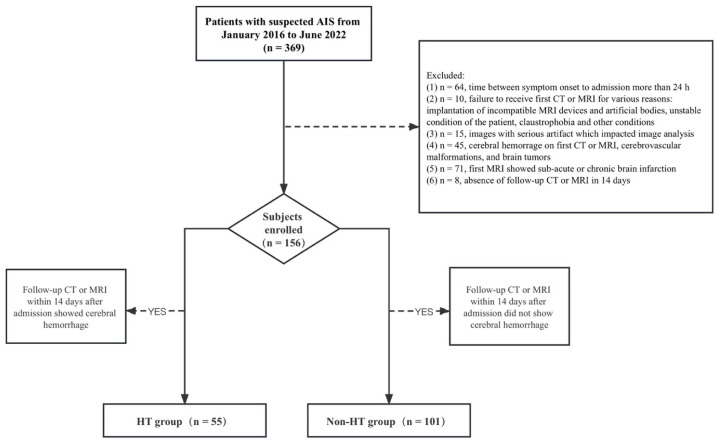
Flow diagram of patient selection.

**Figure 2:**
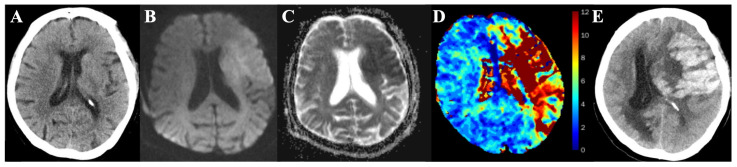
A representative AIS patient with HT in the follow-up CT. An 86-year-old female with right hemiparesis for 2 h. Intravenous thrombolysis therapy was performed after initial CT and MRI. No hemorrhage was found on the initial CT scan (**A**). Initial MR scan showed a patchy ischemic infarct area on the DWI (**B**) and ADC maps (**C**). Tmax map (**D**) showed the infarct area with delayed Tmax. HT was found after thrombolysis. Follow-up CT showed a high density of hemorrhage in the infarct area (**E**).

The flow diagram of detailed patient inclusion and exclusion is shown in Figure 1. A total of 156 patients met the inclusion criteria and were enrolled in this study. The main demographic and clinical information of the patients at baseline are summarized in Table 1. In the 156 AIS patients (mean age 70.9 years ± 12; 102 men) evaluated, HTs were detected in the follow-up radiological examinations in 55 out of 156 patients (35%). Figure 2 illustrates one representative AIS patient with HT in the follow-up CT. All patients were divided into two groups: HT (*n* = 55) and non-HT groups (*n* = 101). The HT group (17 of 55 patients, 30.9%) showed more patients with atrial fibrillation than the non-HT group (9 of 101 patients, 8.9%), with the *p*-value < 0.001. The median time from symptom onset to the first MRI in the HT group (3 h [IQR: 2–5 h]) was shorter than that in the non-HT group (4.5 h [IQR: 3–7 h]), with the *p*-value = 0.01. The median NIHSS score of patients at admission in the HT group was 14 (IQR: 8–19), which is significantly higher than that in the non-HT group (3 [IQR: 2–8]), with a *p*-value < 0.001. A total of 53 out of 55 patients (96.4%) in the HT group received reperfusion therapy, while 42 out of 101 patients (41.2%) in the non-HT group received it (*p* < 0.001). No significant differences were found for age, gender, history of hypertension, and history of diabetes mellitus between the HT and non-HT groups.

### 3.2. MRI Parameters in HT and Non-HT Groups

**Figure 3:**
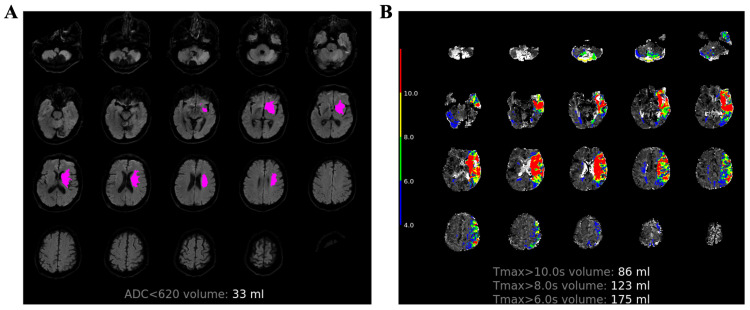
Quantitative perfusion volumes by RAPID. A 76-year-old male with right limb weakness for 2 h who received reperfusion therapy. Volumes of ADC values less than 620 × 10^−6^ mm^2^/s (**A**) and Tmax delays > 6 s, 8 s, and 10 s (**B**) were automatically generated from RAPID software (version 5.1, iSchemaView).

Volumes of ADC values less than 620 × 10^−6^ mm^2^/s and Tmax delays > 6 s, 8 s, and 10 s were automatically generated from RAPID software (version 5.1, iSchemaView), as shown in Figure 3. In the HT group, the median volumes of ADC < 620 × 10^−6^ mm^2^/s and Tmax > 6 s, 8 s and 10 s were 30 (IQR: 8–91) mL, 141 (IQR: 59–190) mL, 98 (IQR: 33–137) mL, and 71 (IQR: 23–102) mL, respectively. In the non-HT group, the median volumes of ADC < 620 × 10^−6^ mm^2^/s and Tmax > 6 s, 8 s, and 10 s were 0 (IQR: 0–8) mL, 0 (IQR: 0–37) mL, 0 (IQR: 0–11) mL, and 0 (IQR: 0–4.5) mL, respectively. As shown in Table 2, significant differences were found for the above four parameters between the HT and non-HT groups, with all the four volumes higher in the HT group (all with *p* < 0.001). 

### 3.3. Multivariable Logistic Regression Analysis of MRI Parameters for Predicting HT

Significant baseline predictors of HT in univariate analyses were atrial fibrillation, time from symptom onset to first MRI, NIHSS score at admission, and reperfusion therapy. As shown in Table 3, in multivariate analyses, after adjusting for the significant baseline predictors, the brain volumes generated from maps of ADC < 620 × 10^−6^ mm^2^/s (OR, 1.018; 95% CI, 1.002–1.035; *p* = 0.024), Tmax > 6 s (OR, 1.018; 95% CI, 1.009–1.028; *p* < 0.001), Tmax > 8 s (OR, 1.027; 95% CI, 1.012–1.042; *p* < 0.001), and Tmax > 10 s (OR, 1.033; 95% CI, 1.013–1.053; *p* = 0.001) all remained as independent predictors of HT. Reperfusion therapy in each separate model remained as an independent predictor of HT (all with *p* < 0.001).

### 3.4. Optimal Cutoff Lesion Volumes of MRI Parameters for Predicting HT

**Figure 4:**
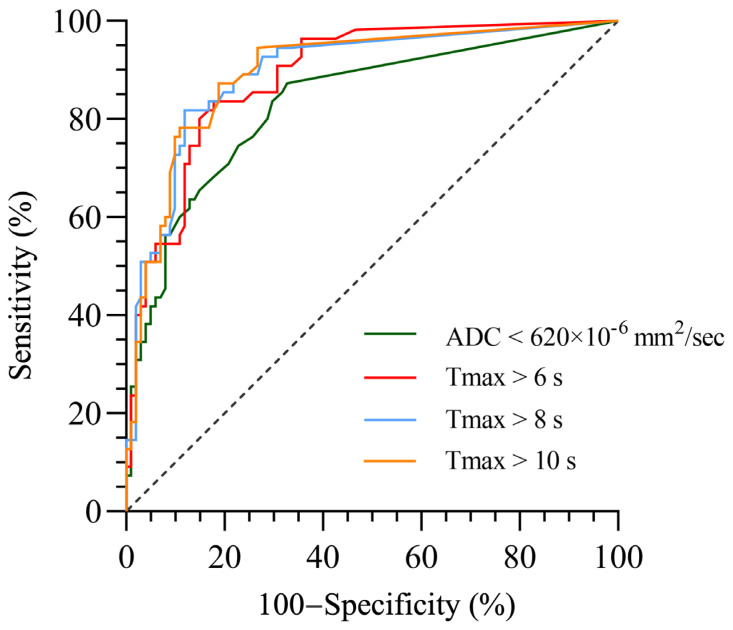
ROC curves of the volumes of ADC < 620 × 10^−6^ mm^2^/s, Tmax > 6 s, Tmax > 8 s, and Tmax > 10 s for predicting HT in AIS patients.

The ROC curve analyses were performed for the four MRI parameters (volumes of ADC < 620 × 10^−6^ mm^2^/s and Tmax > 6 s, 8 s, and 10 s), and Youden indexes were calculated to obtain the proper value of each parameter to predict HT. As shown in Figure 4 and Table 4, the ROC curve analyses of the four MRI parameters demonstrated that the volumes of ADC < 620 × 10^−6^ mm^2^/s (AUC, 0.833; 95% CI, 0.763–0.902), Tmax > 6 s (AUC, 0.889; 95% CI, 0.838–0.941), Tmax > 8 s (AUC, 0.896; 95% CI, 0.842–0.949), and Tmax > 10 s (AUC, 0.898; 95% CI, 0.845–0.950) in predicting HT were significant (all with *p* < 0.001). For the volume of ADC < 620 × 10^−6^ mm^2^/s, the optimal cutoff lesion volume was 2 mL, with a sensitivity of 87.3%, a specificity of 67.3%, and an accuracy of 74.3%. For the volume of Tmax > 6 s, the optimal cutoff lesion volume was 47 mL, with a sensitivity of 83.6%, a specificity of 82.2%, and an accuracy of 82.7%. For the volume of Tmax > 8 s, the optimal cutoff lesion volume was 29.5 mL, with a sensitivity of 81.8%, a specificity of 88.1%, and an accuracy of 85.9%. For the volume of Tmax > 10 s, the optimal cutoff lesion volume was 10.5 mL, with a sensitivity of 87.3%, a specificity of 81.2%, and an accuracy of 83.4%.

## 4. Discussion

AIS occurs when a brain artery is blocked by a blood clot and results in brain tissue hypoperfusion. HT after AIS is a common event, especially after reperfusion therapy. In this study, 35% of all AIS patients suffered from HT within 14 days after symptom onset, which is consistent with previous reports [6,26]. The identification of risk factors for HT provides key information to guide the clinical treatment of AIS patients, which may improve the selection of patients and the safety of treatment. In this retrospective study, we found that atrial fibrillation, NIHSS score at admission, and reperfusion therapy were significantly correlated with HT, consistent with previous reports [27,28,29]. AIS patients’ atrial fibrillations are mostly of cardioembolic origin, due to the emboli falling off. Atrial fibrillation was found to increase the severity of stroke patients with a higher NIHSS score, which may be attributed to the embolic occlusion of larger cerebral arteries [30]. For these patients, doctors may be more likely to conduct reperfusion therapy, which also increases the risk of HT. The underlying mechanism of atrial fibrillation increasing the risk of HT remains to be deeply explored. AIS patients with a higher NIHSS score at admission may have more severe vascular impairments, increasing the risk of HT and impacting long-term prognosis. Reperfusion therapy, including thrombolysis with rt-PA and mechanical thrombectomy, is well known to increase the incidence of HT due to the breakdown of fibrin or the direct damage to the blood vessel walls [31]. However, time from symptom onset to admission was negatively associated with the occurrence of HT in this study, with a shorter time from symptom onset to admission showing more HT. We assumed that the possible reason for this was that more patients with faster admission to the hospital received reperfusion therapy within the treatment window, which can increase the risk of HT. Thus, this result confirmed the finding that the tissue window was more important than the time window, which suggests that there should be a shift from the paradigm of “time is brain” to “imaging is brain” in the management of AIS [32]. Other previously established risk factors, including age, gender, history of hypertension, and history of diabetes mellitus, had no strong associations with HT. 

It has been well known that the volumes of different thresholds of Tmax reflect the ischemic lesions of different degrees of hypoperfusion, and lower ADC values indicate greater degrees of ischemia. Severe hypoperfusion with high Tmax is associated with irreversible neuron death of the infarct areas and poor clinical outcome, even after reperfusion [23]. Our study indicated that the volume of low ADC values was positively related with HT, which is consistent with previous studies that showed that ADC values can be useful for assessing HT [10,11]. However, we found that Tmax > 10 s performed better than ADC in predicting HT. The main finding of our study was that the volume of Tmax threshold > 10 s, generated from PWI-MRI, was the most valuable parameter with the greatest AUC for predicting HT. At this threshold, lesion volume ≥ 10.5 mL optimally predicted HT with a sensitivity of 87.3%, a specificity of 81.2%, and an accuracy of 83.4%. This finding suggests that once the detecting lesion volume ≥ 10.5 mL on Tmax > 10 s on PWI-MRI, the stroke patient may suffer a higher risk of HT, especially after receiving reperfusion therapies of thrombolysis and thrombectomy. PWI-MRI provides significant information with irreplaceable value in the assessment of AIS patients, especially in identifying those who are going to benefit from reperfusion therapies and those who are likely to develop HT after reperfusion therapies.

The underlying mechanism of the phenomenon where the higher volume of Tmax > 10 s relates with a higher risk of HT can be related with multiple factors. BBB disruption has been reported to be an HT driver in AIS [22,33,34]. Stroke-induced BBB disruption facilitates the inflammatory development and progress in the brain, and inflammation promotes the BBB breakdown by disrupting BBB permeability [35,36,37]. The inhibition of myeloperoxidase, a pro-inflammatory enzyme mainly secreted by myeloid cells after stroke in the central nervous system, decreases the inflammatory progress in the brain and improves clinical outcome, as found by John Chen and his colleagues in animal models [38]. Targeting neuro-inflammation can be a potentially effective way to protect neurofunction and reduce HT after restoring brain perfusion in AIS by reducing reactive oxygen species, matrix metalloproteinases, or other inflammatory factors [39,40,41]. Previous studies have shown that MRI-based detection of the BBB integrity assessed by MRI may help predict HT [12,15].

Our study has several limitations. Firstly, the sample size of enrolled patients was relatively small, due to the strict inclusion and exclusion criteria. Secondly, further grouping by different treatment methods of thrombolysis, thrombectomy, or routine anticoagulant therapy was not performed. Thirdly, we did not analyze the risk according to the radiological classification of HT. Future work with an enlarged sample size may overcome the limitations to further investigate the predicting value of PWI.

## 5. Conclusions

In summary, our study demonstrated that the early measurement of the volume on the map of Tmax > 10 s generated from PWI-MRI showed optimal performance for predicting HT, with a cutoff lesion volume of 10.5 mL, among parameters that included ADC value < 620 × 10^−6^ mm^2^/s and Tmax delays > 6 s, 8 s, and 10 s.

## Figures and Tables

**Table 1:** Demographic and clinical information of enrolled patients and the differences between HT and non-HT groups.

Baseline Features	AIS Cases (*n* = 156)	HT Group (*n* = 55)	Non-HT Group (*n* = 101)	*p*-Value
**Demography**				
Age, mean ± SD	70.9 ± 12	71.9 ± 10.9	70.3 ± 12.6	0.433
Male, *n* (%)	102 (65.4%)	34 (61.8%)	68 (67.3%)	0.491
Female, *n* (%)	54 (34.6%)	21 (38.2%)	33 (32.7%)	
**Vascular Risk Factors**				
Hypertension, *n* (%)	96 (61.5%)	37 (67.3%)	59 (58.4%)	0.279
Diabetes mellitus, *n* (%)	37 (23.7%)	13 (23.6%)	24 (23.8%)	0.986
Atrial fibrillation, *n* (%)	26 (16.7%)	17 (30.9%)	9 (8.9%)	<0.001 ^†^
Time of symptom onset (h) *	4 (2.5–6.5)	3 (2–5)	4.5 (3–7)	0.010 ^†^
NIHSS scores at admission *	6.5 (2–14)	14 (8–19)	3 (2–8)	<0.001 ^†^
Reperfusion therapy, *n* (%)	95 (60.1%)	53 (96.4%)	42 (41.6%)	<0.001 ^†^

Note: AIS, acute ischemic stroke; HT, hemorrhagic transformation; SD, standard deviation; NIHSS, National Institutes of Health Stroke Scale. * Numbers are medians, with interquartile ranges in parentheses. ^†^ Indicates *p* < 0.05.

**Table 2:** The differences in MRI parameters between the HT and non-HT groups.

MR Parameters (Volume)	AIS Cases (*n* = 156)	HT Group (*n* = 55)	Non-HT Group (*n* = 101)	*p* Value
ADC < 620 × 10^−6^ mm^2^/s (mL)	5 (0–26.75)	30 (8–91)	0 (0–8)	<0.001
Tmax > 6 s (mL)	26.5 (0–123.75)	141 (59–190)	0 (0–37)	<0.001
Tmax > 8 s (mL)	7 (0–70.75)	98 (33–137)	0 (0–11)	<0.001
Tmax > 10 s (mL)	3 (0–40.5)	71 (23–102)	0 (0–4.5)	<0.001

Note: The results of the four MRI parameters were obtained from RAPID software (version 5.1, iSchemaView). All values were expressed as medians (interquartile ranges). *p*-values were calculated with the Mann–Whitney U test. Abbreviations: ADC, apparent diffusion coefficient; AIS, acute ischemic stroke; HT, hemorrhagic transformation.

**Table 3:** Multivariable logistic regression analysis for predicting HT.

Risk Factors	Regression Coefficient	SD	Wald	OR	95% CI	*p*-Value
Atrial fibrillation	0.483	0.751	0.415	1.621	0.372–7.060	0.520
Time of symptom onset	0.050	0.061	0.681	1.052	0.933–1.185	0.409
NIHSS scores at admission	0.132	0.046	8.200	1.141	1.042–1.248	0.004 *
Reperfusion therapy	3.556	0.892	15.877	35.022	6.091–201.359	<0.001 *
ADC < 620 × 10^−6^ mm^2^/s	0.018	0.008	5.060	1.018	1.002–1.035	0.024 *
Atrial fibrillation	0.457	0.790	0.335	1.580	0.336–7.428	0.562
Time of symptom onset	0.038	0.066	0.327	1.038	0.913–1.181	0.568
NIHSS scores at admission	0.063	0.049	1.627	1.065	0.967–1.173	0.202
Reperfusion therapy	4.652	1.293	12.942	104.776	8.310–1321.082	<0.001 *
Tmax > 6 s	0.018	0.005	13.945	1.018	1.009–1.028	<0.001 *
Atrial fibrillation	0.418	0.804	0.270	1.519	0.314–7.347	0.603
Time of symptom onset	0.050	0.064	0.620	1.051	0.928–1.191	0.431
NIHSS scores at admission	0.064	0.049	1.684	1.066	0.968–1.173	0.194
Reperfusion therapy	4.705	1.322	12.658	110.481	8.273–1475.442	<0.001 *
Tmax > 8 s	0.026	0.007	12.879	1.027	1.012–1.042	<0.001 *
Atrial fibrillation	0.419	0.804	0.271	1.520	0.315–7.344	0.602
Time of symptom onset	0.069	0.061	1.292	1.071	0.951–1.207	0.256
NIHSS scores at admission	0.073	0.048	2.316	1.076	0.979–1.183	0.128
Reperfusion therapy	4.508	1.242	13.177	90.750	7.957–1035.005	<0.001 *
Tmax > 10 s	0.032	0.010	10.675	1.033	1.013–1.053	0.001 *

Note: SD, standard deviation; OR, odds ratio; CI, confidence interval; ADC, apparent diffusion coefficient; NIHSS, National Institutes of Health Stroke Scale. * Indicates *p* < 0.05.

**Table 4:** Volumes of ADC < 620 × 10^−6^ mm^2^/s, Tmax > 6 s, Tmax > 8 s, and Tmax > 10 s optimal values for predicting HT in AIS patients as calculated using ROC curves.

MRI Parameters	AUC (95% CI)	Maximum Youden Index	Cutoff Value (mL)	Sensitivity (%)	Specificity (%)	Accuracy (%)	*p*-Value
ADC < 620 × 10^−6^ mm^2^/s	0.833 (0.763–0.902)	0.546	2.0	87.3	67.3	74.3	<0.001
Tmax > 6 s	0.889 (0.838–0.941)	0.658	47.0	83.6	82.2	82.7	<0.001
Tmax > 8 s	0.896 (0.842–0.949)	0.699	29.5	81.8	88.1	85.9	<0.001
Tmax > 10 s	0.898 (0.845–0.950)	0.685	10.5	87.3	81.2	83.4	<0.001

Note: ADC, apparent diffusion coefficient; AUC, area under curve.

## Data Availability

The datasets generated during the current study are available from the corresponding author upon reasonable request.
